# Role of rhesus macaque IFITM3(2) in simian immunodeficiency virus infection of macaques

**DOI:** 10.1371/journal.pone.0224082

**Published:** 2019-11-04

**Authors:** Michael Winkler, Sabine Gärtner, Lara Markus, Markus Hoffmann, Inga Nehlmeier, Michael Krawczak, Ulrike Sauermann, Stefan Pöhlmann

**Affiliations:** 1 Infection Biology Unit, German Primate Center–Leibniz Institute for Primate Research, Göttingen, Germany; 2 Faculty of Biology and Psychology, University Göttingen, Göttingen, Germany; 3 Institute of Medical Informatics and Statistics, Kiel University, Kiel, Germany; 4 Infection Models Unit, German Primate Center—Leibniz Institute for Primate Research, Göttingen, Germany; University of Pittsburgh, UNITED STATES

## Abstract

The experimental infection of rhesus macaques (rh) with simian immunodeficiency virus (SIV) is an important model for human immunodeficiency virus (HIV) infection of humans. The interferon-induced transmembrane protein 3 (IFITM3) inhibits HIV and SIV infection at the stage of host cell entry. However, it is still unclear to what extent the antiviral activity of IFITM3 observed in cell culture translates into inhibition of HIV/SIV spread in the infected host. We have shown previously that although rhIFITM3 inhibits SIV entry into cultured cells, polymorphisms in the *rhIFITM3* gene are not strongly associated with viral load or disease progression in SIV infected macaques. Here, we examined whether rhIFITM3(2), which is closely related to rhIFITM3 at the sequence level, exerts antiviral activity and whether polymorphisms in the *rhIFITM3(2)* gene impact the course of SIV infection. We show that expression of rhIFITM3(2) is interferon-inducible and inhibits SIV entry into cells, although with reduced efficiency as compared to rhIFITM3. We further report the identification of 19 polymorphisms in the *rhIFITM3(2)* gene. However, analysis of a well characterized cohort of SIV infected macaques revealed that none of the polymorphisms had a significant impact upon the course of SIV infection. These results and our previous work suggest that polymorphisms in the *rhIFITM3* and *rhIFITM3(2)* genes do not strongly modulate the course of SIV infection in macaques.

## Introduction

The interferon (IFN) system is an integral component of innate immunity and constitutes the first line of defense against viral infection. The antiviral effect of the IFN response is due to the IFN-induced expression of approximately 400 genes, many of which encode proteins that exert antiviral activity [1, 2]. The IFN-induced transmembrane protein 3 (IFITM3) was identified in a screen for host cell factors modulating influenza A virus (FLUAV) infection [3] and inhibits host cell entry of FLUAV and several other viral pathogens [3–5]. Inhibition of viral entry is believed to occur at the stage of hemifusion or fusion pore formation and may entail alterations of the biophysical properties of membranes [6, 7], which in turn may require IFITM/IFITM interactions [8]. Importantly, intact IFITM3 is essential for defense against severe influenza [9, 10], indicating the protein exerts potent antiviral activity in the infected host.

Several lines of evidence suggest that IFITM3 might impact HIV/SIV infection. IFITM3 was shown to inhibit host cell entry of HIV and SIV [11, 12] and to be incorporated into viral particles, which reduces viral infectivity [13–15]. Moreover, evidence has been provided that IFITM3 can interfere with the processing of the viral envelope protein (Env) by host cell proteases, which is essential for viral infectivity [16]. Finally, it has been demonstrated that transmitter-founder viruses, which successfully trespass the mucosal barrier and establish HIV infection upon sexual transmission, are highly resistant against inhibition by IFITM3 [17]. The same study also showed that IFITM3 is important for IFN-induced inhibition of HIV infection of cultured primary cells [17]. Collectively, these results indicate that IFITM3 might pose a barrier against sexual transmission of HIV/SIV and might modulate viral spread in the infected host. However, direct proof of a role of IFITM3 in HIV/SIV amplification in the host and in disease progression is still lacking.

**Fig 1:**
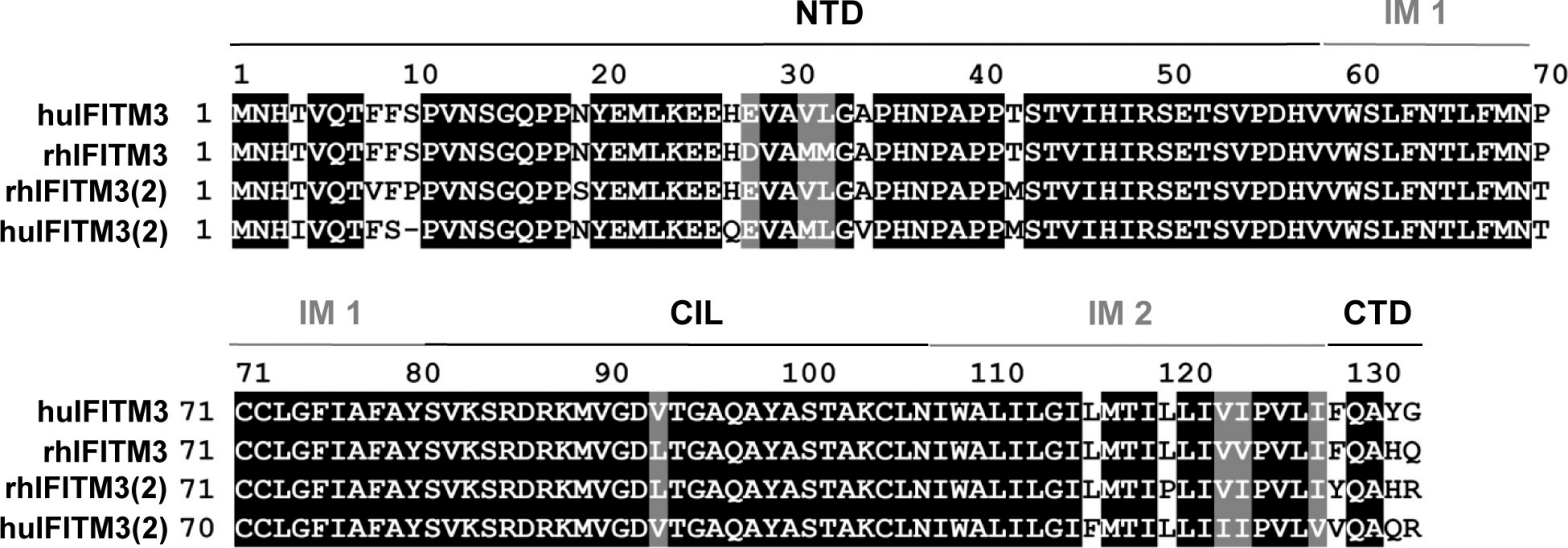
Amino acid alignments of huIFITM3, huIFITM2, rhIFITM3 and rhIFITM3(2). Alignments were prepared using Clustal W (Vector NTI AlignX) and output formatted using BoxShade (https://embnet.vital-it.ch/software/BOX_form.html). Identical residues are shown with black background, while nonidentical but similar residues are shown with gray background. NTD, N-terminal domain; IM, intermembrane domain; CIL, conserve intracellular loop; CTP, C-terminal domain.

We have shown previously that the rhesus macaque (rh) homologue of human (hu) IFITM3 inhibits SIV entry into transfected cells and we identified 16 polymorphisms in the *rhIFITM3* gene, three of which were located in exons [18]. However, none of the 16 polymorphisms significantly modulated peak viral load or disease progression in macaque cohorts experimentally infected with SIV [18]. In the course of our studies, we also identified a rhIFITM3 homologue, termed rhIFITM3(2). The *rhIFITM3(2)* and *rhIFITM3* genes are both located on chromosome 14 and encoded proteins share 91% sequence identity (Fig 1). However, rhIFITM3(2) exhibits two amino acid differences in the highly conserved central part that are also found in huIFITM2, and the protein may thus differ from rhIFITM3 in antiviral activity (Fig 1). Whether rhIFITM3(2) inhibits SIV infection is presently unknown.

Here, we show that expression of rhIFITM3(2) is IFN-inducible and inhibits SIV Env-driven entry into target cells, although with modest efficiency. However, polymorphisms in the *rhIFITM3(2)* gene were not found to strongly impact the course of SIV infection.

## Materials and methods

### Cells and plasmids

Human embryonal kidney 293T (DSMZ ACC 635) and the rhesus macaque cell lines sMAGI (mammary tumor, [19]), TeloRF (telomerase-immortalized fibroblasts, [20]), LLC-MK2 (kidney epithelium, [21]) and MaMuK/8639 (kidney) were cultivated in Dulbeccos Modified Eagles Medium (DMEM), supplemented with 10% fetal calf serum (FCS), L-glutamine and penicillin/streptomycin, at 37°C in a humidified atmosphere with 5% CO_2_. sMAGI, TeloRF and LLC-MK2 cells were obtained from collaborators, MaMuK/8639 cells were generated in the course of the present study. Plasmids encoding murine leukemia virus envelope protein (MLV-Env), SIV envelope proteins (SIVmac239 Env) and FLUAV strain A/WSN/33 hemagglutinin (HA) and neuraminidase (NA) have been described previously [22–25] To generate an expression plasmid for SIVmac316 Env an XhoI/EcoRI fragment, containing env and 3’LTR sequences, was subcloned from proviral DNA into pcDNA3.1(-) (Invitrogen). The MLV-based retroviral vector pQCXIP encoding human and macaque IFITM3 proteins (huIFITM3, rhIFITM3, rhIFITM3(2)) or chloramphenicol acetyltransferase (Cat) as control as well as MLV-luc, MLV-gag-pol and VSV-G expression plasmids have been reported previously [3, 23, 26–28]. A mutated version of rhIFITM3(2) carrying the T70S mutation was generated by splice overlap PCR using primers MamuIFITM3(2)-5NA (5-CCCGCGGCCGCACCGGTACCATGAACCACACGGTCCAAAC-3)/ mut-mmIF3-2-T70S-rev (5- AATGCTATGAAGCCTAGGCAGCAGGAGTTCATGAAGAGGG-3) and mut-mmIF3-2-T70S-for (5- CCCTCTTCATGAACTCCTGCTGCCTAGGCTTCATAGCATT-3)/MamuIFITM3(2)-3E (5- CGAATTCCTATCGATGGGCTTGGTAGATC-3) and pQCXIP-rhIFITM3(2) as template. The final PCR product was digested with NotI and EcoRI and cloned into pQCXIP-mcs [29]. The identity of all PCR-amplified sequences was verified using automated sequencing.

### Production of MLV particles

A published protocol was used for the generation of MLV particles encoding IFITM or Cat proteins for transduction experiments [25, 26]. Briefly, 293T cells (625,000 cells/T25 flask) were cotransfected with 6 μg pQCXIP vector harboring IFITM genes or Cat, 3 μg MLV-gag-pol and 3 μg VSV-G plasmids. Medium was changed after 6 h and supernatants harvested at 48 h post transfection, passed through 0.45 μm filters, aliquoted and stored at -80°C. MLV reporter particles pseudotyped with different viral Env proteins for analysis of entry inhibition by IFITM proteins were produced in the same way, using 6 μg MLV-luc, 3 μg MLV-gag-pol and 3 μg of the respective envelope-encoding plasmid. For particles bearing FLUAV surface proteins, 2 μg of each plasmid, HA and NA, was used. The MLV luc plasmid contains a HCMV enhancer/promoter MLV-LTR (R-U5) control region followed by a VL30 ‘‘gag-less” packaging signal and a full 3’-LTR (U3-R-U5). The LTR elements were derived from Friend murine leukemia virus FB29. The firefly luciferase cDNA under control of the HCMV enhancer/promoter was inserted between packaging signal and 3’LTR.

### Analysis of the antiviral activity of IFITM proteins

The antiviral activity of IFITM proteins was assessed as described previously [26, 27]. Briefly, 293T cells were seeded in 96-well plates at 10^4^ cells per well in 50 μl medium. The next day, 50 μl of transducing particles were added per well followed by spinoculation [30] at 4,000×g for 30 min. After two days, the medium was replaced by 50 μl fresh cell culture medium, followed by transduction with 50 μl of MLV particles harboring the viral glycoproteins to be analyzed for IFITM sensitivity and normalized for equal luciferase activity upon transduction of control cells. After incubation for 8 h, the medium was replaced by fresh cell culture medium. Luciferase activities in cell lysates were analyzed at 72 h post infection in a microplate reader (Plate Chameleon V, Hidex, Turku, Finland) employing commercial Beetle -Juice kit (PJK) kits.

### Animals

A cohort of 39 SIV-infected macaques (Macaca mulatta) of Indian origin was screened retrospectively for *rhIFITM3(2)* polymorphisms. The same cohort was used previously to search for polymorphisms modulating AIDS-free survival time and viral load in SIV infection [18, 31]. Macaques were either bred at the German Primate Center and had known ancestry, or originated from breeders in France, United Kingdom or the United States. For three animals, PCR-amplification of *rhIFITM3(2)* was not successful; genetic and phenotypic data for the remaining 36 animals are summarized in Table 1. Animals were infected intravenously, intrarectally or via tonsils with a single dose of SIVmac239, SIVmac251 or SIVmac239/32H and displayed the whole spectrum of disease progression, from rapid progression to long-term non-progression, for each combination of virus and infection route. The definition of early AIDS-defining illness was based upon clinical, necropsy and histopathological findings as described [31].

**Table 1:** Description of the cohort.

No of macaques	47
**Sex (%)**	
Male	36 (76.6)
Female	11 (23.4)
**Infecting SIVmac (%)**	
251	16 (34)
251/32H	13 (27.6)
251/32H/ex vivo, spleen	3 (6.4)
239	15 (32)
**Route of infection (%)**	
Intravenous	29 (61.7)
Intrarectal	5 (10.6)
Tonsillar	13 (27.7)
**Median age (years) at infection**	4.4 (2–9)
**Mean survival (weeks) after infection**	132 (6–907)
**Death with AIDS-related symptoms (%)**	34 (72.3)
Mamu-A1*001 positive (%)	10 (21.2)

### Ethics statement

All animals were housed at the German Primate Center (DPZ) under conditions in accordance with the German Animal Protection Act and the European Union guidelines on the use of non-human primates for biomedical research, as described [32]. Experiments were approved by an external ethics committee authorized by the Lower Saxony State Office for Consumer Protection and Food Safety (project licenses: 509.42502/08-02.95, 509.42502/08-13.98, 504.42502/08-03.90 (V+Ä), 33.9-42502-04-12/0820, 509-42502/08-04.03, 33.9.42502/04/017/07, 33.9.42502/04/72/08). According to §11 of the German Animal Welfare Act, the DPZ is permitted to breed and house non-human primates under license number 392001/7 issued by the local veterinary authorities. All macaques were under daily surveillance by veterinarians and animal caretakers. During quarantine, the animals were usually housed in groups of 2–3 animals per cage, with a minimum enclosure height of 180 cm and a cage volume of 3 to 4.5 m^3^. During experiments, animals had to be housed singly (cage dimensions [in cm]: 190h × 90w × 90d), but with constant visual, olfactory and acoustic contact to their roommates. Procedures for animal welfare and to minimize discomfort and suffering were undertaken in accordance with the recommendations of the Weatherall report (“The use of nonhuman primates in research”) [33]. Monkeys were fed with dry monkey biscuits supplemented with fresh fruit or vegetables twice daily and with fresh water access ad libitum. For environmental enrichment, animals were offered feeding puzzles, varying toys and wood sticks for gnawing. In addition, music was played. For DNA preparation, blood samples were drawn under anesthesia with 10 mg ketamine i.m. per kg body weight. Animals were humanely euthanized by an overdose of Pentobarbital-Natrium (Narcoren1, Merial, Hallbergmoos, Germany) under anesthesia either for experimental reasons without exhibiting clinical symptoms or in cases of suffering predefined by a scoring system of termination criteria that was approved by the external ethics committee and corresponds to the IACUC endpoint guidelines, as described [32]. For the purpose of this study, no monkey was sacrificed since archival samples were used.

### DNA sequence analysis of *rhIFITM3(2)*

The *rhIFITM3(2)* gene (LOC697564) was amplified from genomic DNA by PCR and screened for polymorphisms by sequencing. Primers for amplification were based upon the Macaca mulatta chromosome 14 scaffold (Mmul_051212) [34], which had been used successfully before in the amplification of cDNAs of *rhIFITM3* genes [27]. Amplification of *rhIFITM3(2)* (LOC697564) was performed with primers mamuIFITM3gen5-1forA (5-TTTGTTCCGCCCTCATCTGG-3) and MamuIFITM3(2)gen3 (5- CTAAGGCAAGAACCTTTGGCG-3). Specific amplification of *rhIFITM3(2)* sequences by this primer pair was confirmed by sequence anlaysis. Since the forward primer was identical to that used for amplification of the *rhIFITM3* gene, we also tested a different forward primer, mamuIFITM3(2)gen5-1for (5-CTGGCCCCGGCCAACTCTGC-3), which contains several mismatches to the rhIFITM3 gene, as forward primer. However, sequencing results of mamuIFITM3(2)gen5-1for/MamuIFITM3(2)gen3 showed no difference to the mamuIFITM3gen5-1forA/MamuIFITM3(2)gen3 amplification, indicating high specificity. Reaction mixtures contained 100 ng genomic DNA, 50 pmol of each primer, 100 μM dNTP mix, 10% betain, 1x HF synthesis buffer and 1 U Phusion DNA polymerase (ThermoFisher). PCR cycling started with 5 min denaturation followed by 40 cycles of denaturation (30 s at 95°C), annealing (30 s at 68°C) and elongation (1 min 40 s at 72°C) and concluded with 10 min elongation at 72°C and cooling to 12°C. Amplification products were separated on 1% agarose gels, purified and sequenced directly. Primers mamuIFITM3gen5-1forA, MamuIFITM3(2)gen3, mamuIFITM3(2)-212 (5-GAGATGCTCAAGGAAGAGC-3), mamuIFITM3(2)-d532 (5-GTGTGCCCAGTAGCTGTC-3), mamuIFITM3-d532 (5-CCCGTGTGTGCCCACGTC-3), mamuIFITM3(2)-539for (5-GTCAGTAGCTTTGTCTGTGT-3), mamuIFITM3(2)-830rev (5-CAGGCTCAGACTCCCCAGG-3), mamuIFITM3(2)-849for (5-GTGAGGAAGGGGAGGAGGTG-3), mamuIFITM3(2)-867for (5-TGGTCCCTGATCTCAGAGTG-3), mamuIFITM3(2)-1209for (5-GCTGATACAGGACTCAGCT-3), mIF3-1495AG (5-GAGTGAGGAAGGGGAGAG-3) were used for sequencing of both strands. Amplicons were assembled on a scaffold sequence using Vector NTI ContigExpress and polymorphisms were identified by visual inspection.

### Analysis of ISG transcript numbers by quantitative PCR

Total cellular RNA of sMAGI, MaMuK/8639, TeloRF and LLC-MK2 cells that were either control-treated or treated for 24 h with 100 U/ml pan-IFNα- (Universal Type I Interferon, PBL Assay Science) was extracted using the RNeasy Mini Kit (Qiagen, Hilden, Germany) following the manufacturer’s instructions. Next, 1 μg RNA was treated with DNase I (New England Biolabs, Ipswich, MA, USA) and used for cDNA synthesis using the SuperScript III First-Strand Synthesis System (ThermoFisher Scientific, Waltham, MA, USA) following the manufacturer’s instructions for the Oligo-dT strategy. Finally, quantitative PCR was performed employing the QuantiTect SYBR Green PCR Kit (Qiagen, Hilden, Germany). For this, separate reactions including 0.5 μl of cDNA (total volume after cDNA synthesis = 20 μl) and primers targeting ß-actin (housekeeping gene control), MX1 (myxovirus resistance protein), ISG56 (interferon-stimulated gene 56), rhIFITM3 or rhIFITM3(2) were prepared (primer sequences are available upon request) and analyzed on a Rotor-Gene Q platform (Qiagen, Hilden, Germany). Induction of *MX1*, *ISG56*, *rhIFITM3* and *rhIFITM3(2)* gene expression following stimulation with IFN was analyzed by the 2^-ΔΔ^Ct-method [35] with ß-actin as reference gene and is displayed as expression fold change.

### Immunoblot analysis of rhIFITM3(2) expression

For the analysis of rhIFITM3(2) protein expression, 293T cells were seeded in 24 well plates at 10^5^ cells per well in 500 μl medium. The next day, 500 μl of transducing particles were added per well followed by spinoculation at 4,000×g for 30 min and subsequent replacement with fresh medium. After two days, medium was removed and cells were lysed by adding 100 μl SDS lysis buffer (1% SDS, 2.5% ß-mercaptoethanol, 5% glycerine, 0.5% bromophenol blue, 0.5mM EDTA, 0.5M Tris pH 6.8) to each well. Samples were heated to 95°C for 10 min and separated on 17.5% polyacrylamide gels. Subsequently, proteins were blotted onto nitrocellulose membranes (GE Healthcare) in a Mini-PROTEAN Tetra Cell (BioRad) at 110 V for 90 minutes. Membranes were blocked with 5% skimmed milk diluted in PBS/0.5% Tween for 1 h at room temperature. Primary antibodies were added in PBS/5% skimmed milk/0.5% Tween and incubated over night at 4°C. Polyclonal rabbit anti-human IFITM2 (1:5,000; Proteintech Group) and mouse monoclonal anti-ß-actin (1:1,000; Sigma) were used as primary antibodies. After washing in PBS/0.5% Tween at room temperature for 3×10 min, the membranes were incubated with anti-rabbit or anti-mouse HRP (horseradish peroxidase)-conjugated secondary antibodies (1:10,000; Dianova) for one hour. After washing three times in PBS/0.5% Tween protein bands were detected using a chemiluminescent substrate (HRP juice plus, P.J.K.) and visualized using a ChemoCam imager (INTAS).

### Statistical analysis

AIDS-free survival analysis was performed using the LIFETEST procedure of the SAS software version 9.4 (SAS Institute Inc., Cary, NC). Virus load association with *rhIFITM3(2)* polymorphisms was assessed for statistical significance by a Mann-Whitney test (two genotypes) or a Kruskal-Wallis test (three genotypes), as appropriate, using Graphpad Prism software (Graphpad, La Jolla, USA). All p-values were two-sided and the level of statistical significance was marked as follows, *, p<0.05; **, p<0.01; ***, p<0.001.

## Results

### Rhesus macaque IFITM3(2) exerts antiviral activity

**Fig 2:**
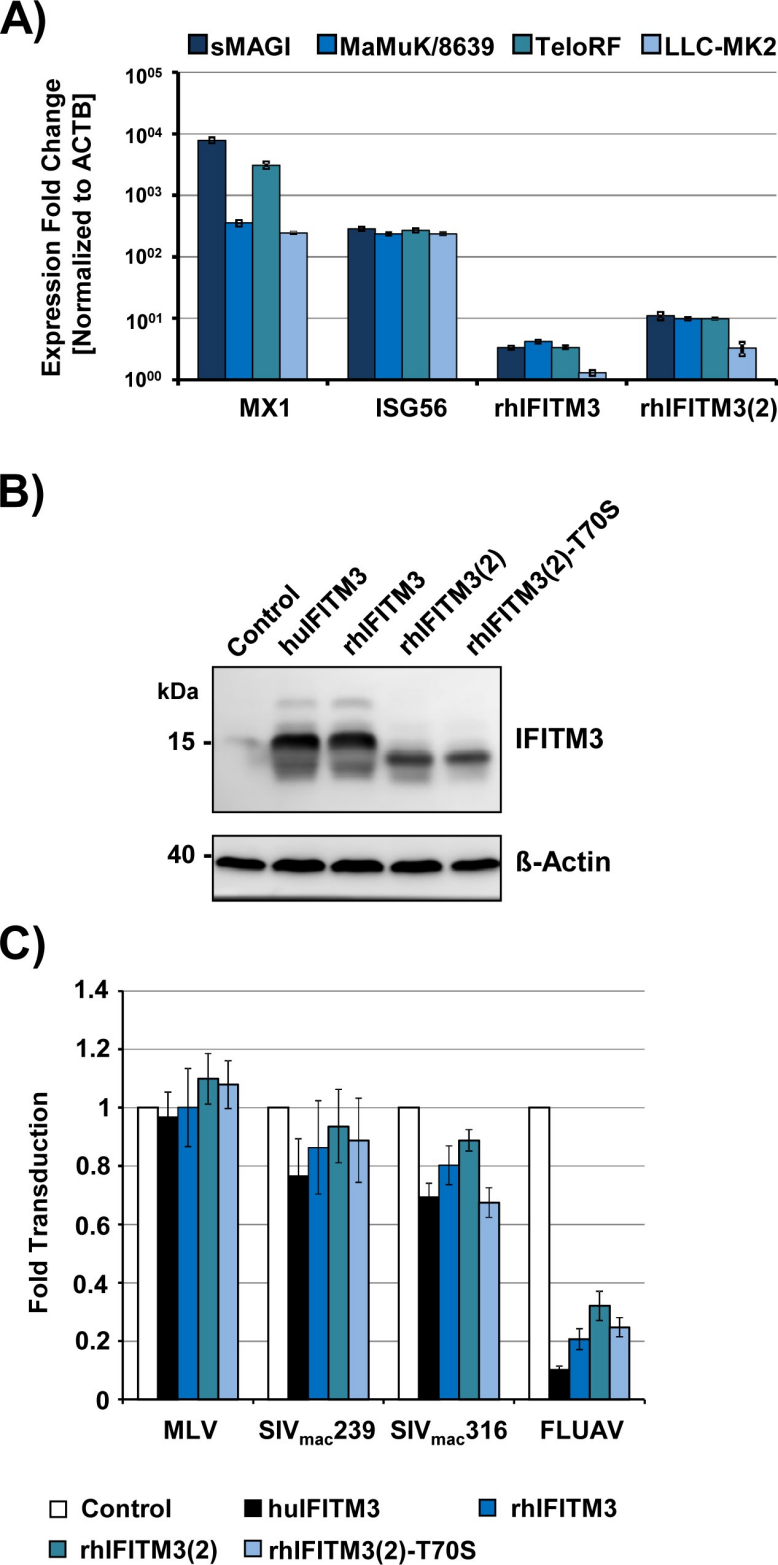
rhIFITM3(2) exerts antiviral activity. (A) The indicated rhesus macaque cell lines were incubated in the presence of pan-IFNα (IFN, 100 U/ml, 24 h) or control-treated, before transcript levels for *ß-actin* (*ACTB*), *MX1*, *ISG56*, *rhIFITM3* and *rhIFITM3(2)* were analyzed by quantitative RT-PCR and normalized against transcript levels for ß-actin (reference gene). Differences in the relative number of *MX1*, *ISG56*, *rhIFITM3* and *rhIFITM3(2)* transcripts between IFN-stimulated and control (mock)-treated cells were calculated by the 2^-ΔΔ^Ct-method [35] including normalization against *ACTB (*housekeeping gene control) levels, and are shown as expression fold change. The results of a single experiment performed with triplicate samples are shown and were confirmed in a separate experiment. Error bars indicate the standard deviation. (B) 293T cells were transduced to express the indicated IFITM proteins and expression was detected by immunoblot using a polyclonal antibody raised against human IFITM2. Expression of β-actin served as loading control. Similar results were obtained in three independent experiments. (C) 293T cells were transfected to express rhesus macaque CD4 and CCR5 genes and then transduced to express either control (chloramphenicol-acetyltransferase, Cat), human (hu) and rhesus macaque (rh) IFITM3 or rhIFITM3(2) genes as indicated. Cells were subsequently transduced with MLV particles encoding luciferase and bearing the indicated viral glycoproteins. Luciferase activities in cell lysates were determined at 72 h post transduction. The average of six independent experiments is shown. Luciferase activities measured in control cells were set as 1. Error bars indicate standard error of the mean.

We first assessed whether *rhIFITM3(2)* is an IFN-induced gene and whether rhIFITM3(2), like huIFITM3 and rhIFITM3, is able to block SIV entry into target cells. To this end, we incubated four rhesus macaque cell lines, sMAGI, MaMuK/8639, TeloRF and LLC-MK2 with pan-IFNα and measured expression of *huIFITM3*, *rhIFITM3* and *rhIFITM3(2)* by qRT-PCR. We found that pan-IFNα treatment markedly stimulated expression of two known IFN-stimulated genes, *MX1 and ISG56*, by at least 100-fold (Fig 2A). Expression of *rhIFITM3* and *rhIFITM3(2)* was also increased upon IFN treatment but induction of expression ranged from 3-fold (*rhIFITM3*) to 10-fold (*rhIFITM3(2)*). Thus, expression of *rhIFITM3(2)* is IFN-inducible and might contribute to control of virus infection. Therefore, we next studied whether rhIFITM3(2) can interfere with host cell entry driven by the SIV-Env protein. We transfected 293T cells to express the SIV receptor CD4 and the coreceptor CCR5 and then transduced the cells with vectors encoding human or rhesus macaque IFITM proteins. Subsequently, we transduced the cells with luciferase-encoding murine leukemia virus (MLV) particles bearing SIV-Env. In parallel, we examined particles harboring the Env protein of MLV or the hemagglutinin (HA) of FLUAV, because it is well known that MLV Env-driven entry is largely resistant to inhibition by IFITM3 while FLUAV-HA-mediated entry is highly sensitive [3, 4].

Immunoblot analysis revealed that all IFITM3 proteins analyzed were expressed in the transduced cells although relative expression was higher for huIFITM3 and rhIFITM3 than for rhIFITM3(2) (Fig 2B, S1 Fig). Why rhIFITM3 and rhIFITM3(2) exhibited a slightly different gel migration is presently unknown but might reflect differential posttanslational processing. Expression of huIFITM3 had no impact on transduction mediated by MLV-Env but markedly reduced transduction driven by HA of FLUAV strain A/WSN/33 (Fig 2C), as expected [3]. Moreover, huIFITM3 reduced transduction mediated by the Env proteins of SIVmac239 and its macrophage tropic variant SIVmac316 by about 30%, in agreement with previous studies [12–14, 16, 18] (Fig 2C). RhIFITM3 also diminished SIV-Env and FLUAV-HA-driven entry, although with reduced efficiency compared to huIFTIM3, in keeping with our previous findings [18]. Finally, rhIFITM3(2) showed lower but detectable inhibitory activity compared to rhIFITM3 (Fig 2C), potentially due to the reduced expression of the former (Fig 2B). Collectively, these results indicate that *rhIFITM3(2)* expression is IFN-inducible and that the protein exerts antiviral activity against SIV.

### Identification of polymorphisms in rhIFITM3(2)

**Fig 3:**
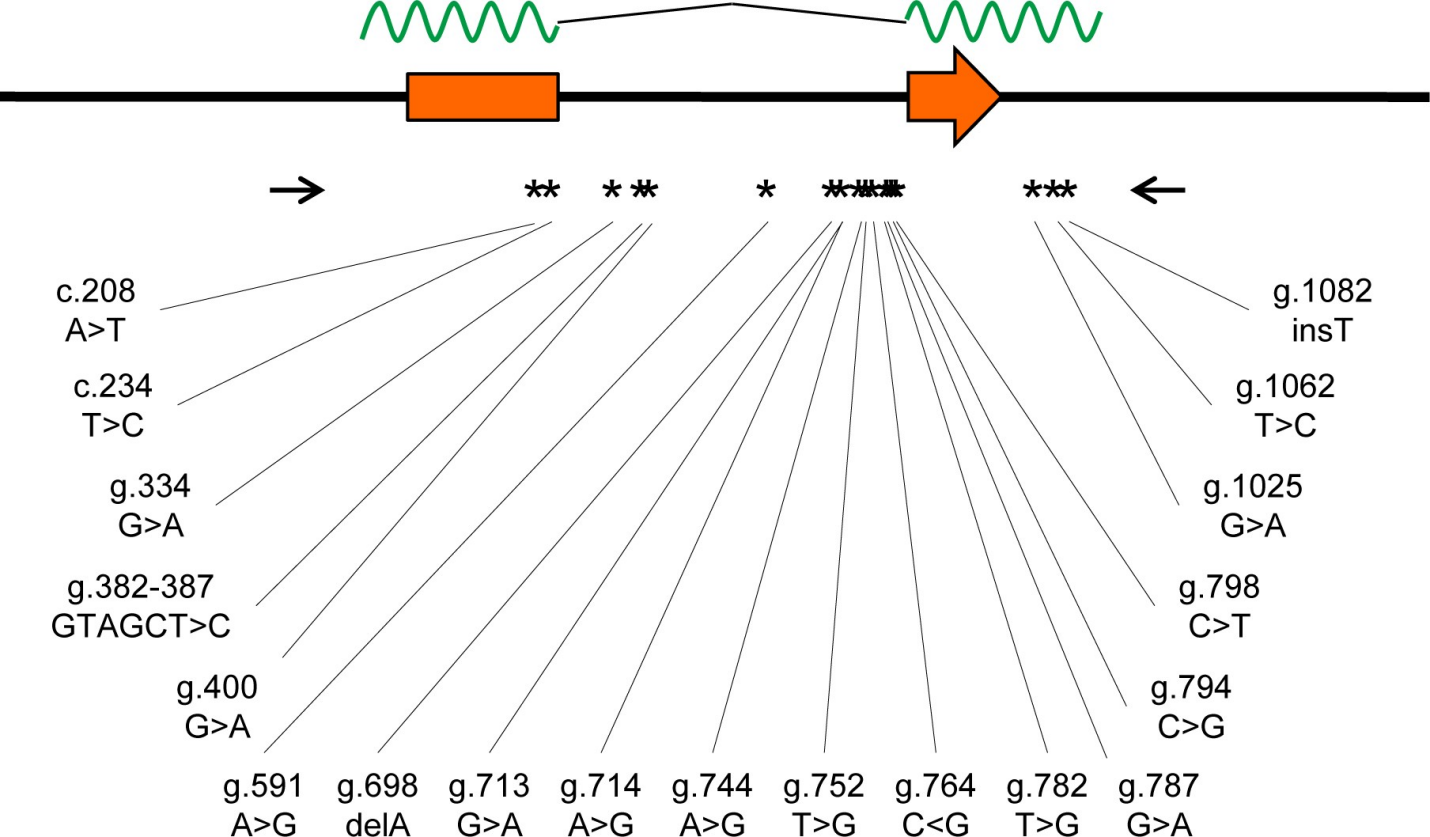
Overview of the *Macaca mulatta IFITM3(2)* gene locus and newly identified sequence variation. The *Macaca mulatta IFITM3(2)* gene locus is shown based upon assembly Mmul_051212. Exons are indicated as orange rectangles with an arrow giving the direction of translation. The mRNA is indicated above as a green wavy line, with the intron represented by a black line. The positions of the primers used for amplification are shown as arrows below the *IFITM3(2)* locus. The positions of polymorphisms are marked by stars and the exact nucleotide position (relative to the start codon) and the detected variations are given.

Next, we investigated whether *rhIFITM3(2)* was polymorphic in a previously characterized macaque colony comprising 47 animals for which pairwise blood relatedness and other confounding factors had been minimized (Table 1). The *rhIFITM3(2)* gene is localized on chromosome 14 and contains two exons that give rise to a predicted mRNA of 641 nts (Genbank entry XM_001085331). For the analysis of the *rhIFITM3(2)* sequence, a PCR strategy was followed that amplified 1360 bp covering the whole transcribed region. Subsequently, PCR products were directly subjected to sequence analysis, which identified 19 polymorphisms (Fig 3). Two of the polymorphisms were located in the coding region of exon 1, three were present in the non-coding region of exon 2 and the remaining polymorphisms were intronic (Fig 3). Most of the polymorphisms located in the non-coding regions were single nucleotide substitutions but insertion and deletion of single bases and, in one instance, five bases were also observed (Table 2). One of the two polymorphisms located in the coding region resulted in an amino acid substitution, T70S. It affected one of the two positions within the conserved central part of IFITMs where rhIFITM3(2) is identical to huIFITM2 but differs from huIFITM3 (which codes for proline at this position). Heterozygosity and major allele frequency were usually both between 0.25 and 0.75, respectively (Table 2). Only one polymorphism (g.1062 T>C) had a low heterozygosity (<10%) and three had a major allele frequency of > 0.90, including c.208 A>T that causes the T70S amino acid exchange.

**Table 2:** Frequency of polymorphisms.

*Position*	*Mutation*	*Major Allele Frequency*	*Observed Heterozygosity*
c.208 A>T	coding, T70S	0.91	0.17
c.234 T>C	coding, silent	0.91	0.17
g.334 G>A	non-coding	0.64	0.26
g.382-387 GTAGCT>C	non-coding	0.64	0.26
g.400 G>A	non-coding	0.64	0.26
g.591 A>G	non-coding	0.77	0.26
g.698 delA	non-coding, deletion	0.59	0.45
g.713 G>A	non-coding	0.56	0.45
g.714 A>G	non-coding	0.56	0.45
g.744 A>G	non-coding	0.57	0.43
g.752 T>G	non-coding	0.64	0.26
g.764 C>T	non-coding	0.64	0.26
g.782 T>G	non-coding	0.64	0.43
g.787 G>A	non-coding	0.64	0.26
g.794 C>G	non-coding	0.64	0.26
g.798 C>T	non-coding	0.64	0.43
g.1025 G>A	non-coding	0.64	0.26
g.1062 T>C	non-coding	0.93	0.06
g.1082 insT	non-coding	0.65	0.23

### Rhesus macaque IFITM3(2) polymorphisms in SIV infection

**Fig 4:**
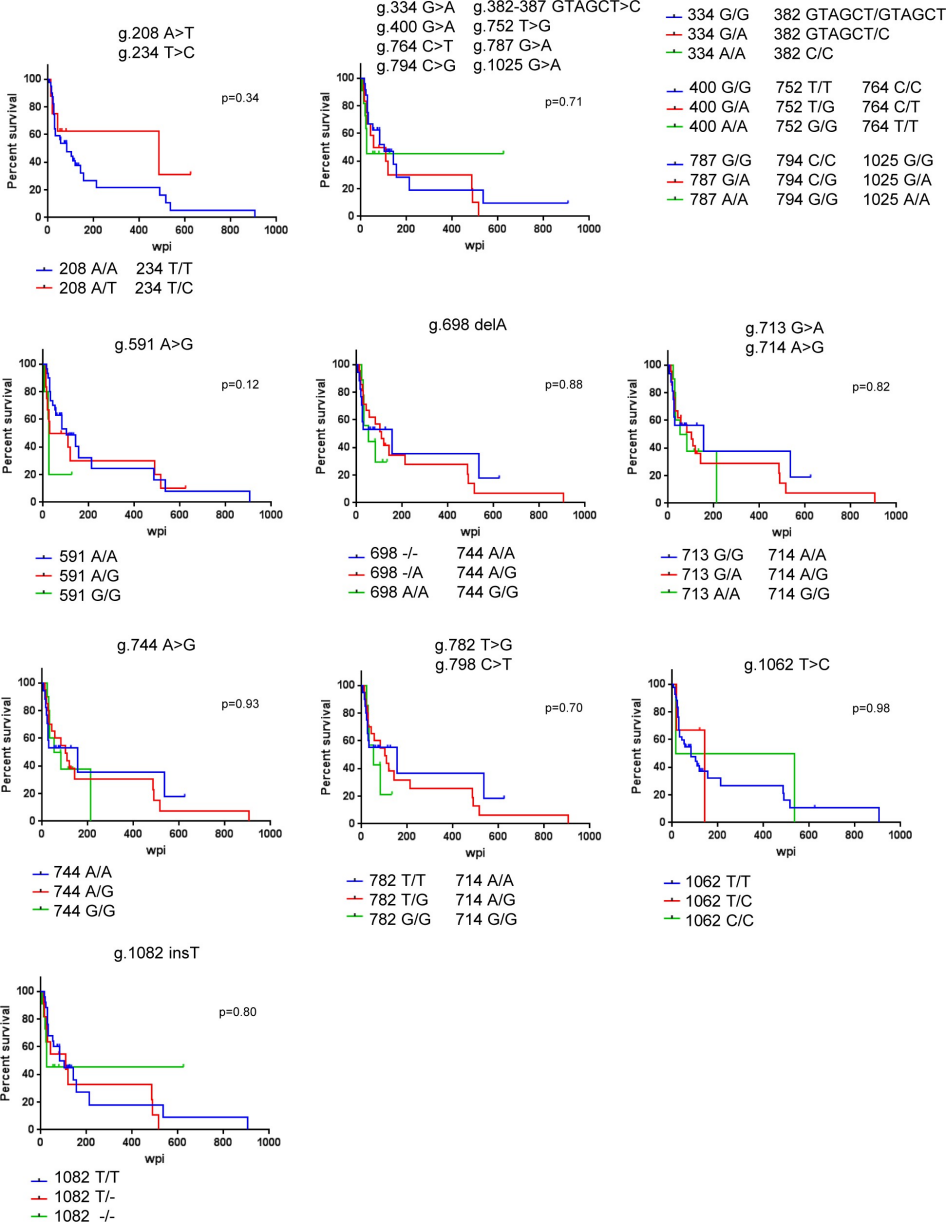
*rhIFITM3(2)* alleles and disease progression in SIV-infected rhesus macaques. For each polymorphism, the AIDS-free survival (percent survival) is depicted by a Kaplan-Meier plot. Censored observations are marked as black ticks. The polymorphisms are indicated above each diagram together with the p-values obtained by a log-rank test. AIDS-free survival time is shown as weeks post infection (wpi).

**Fig 5:**
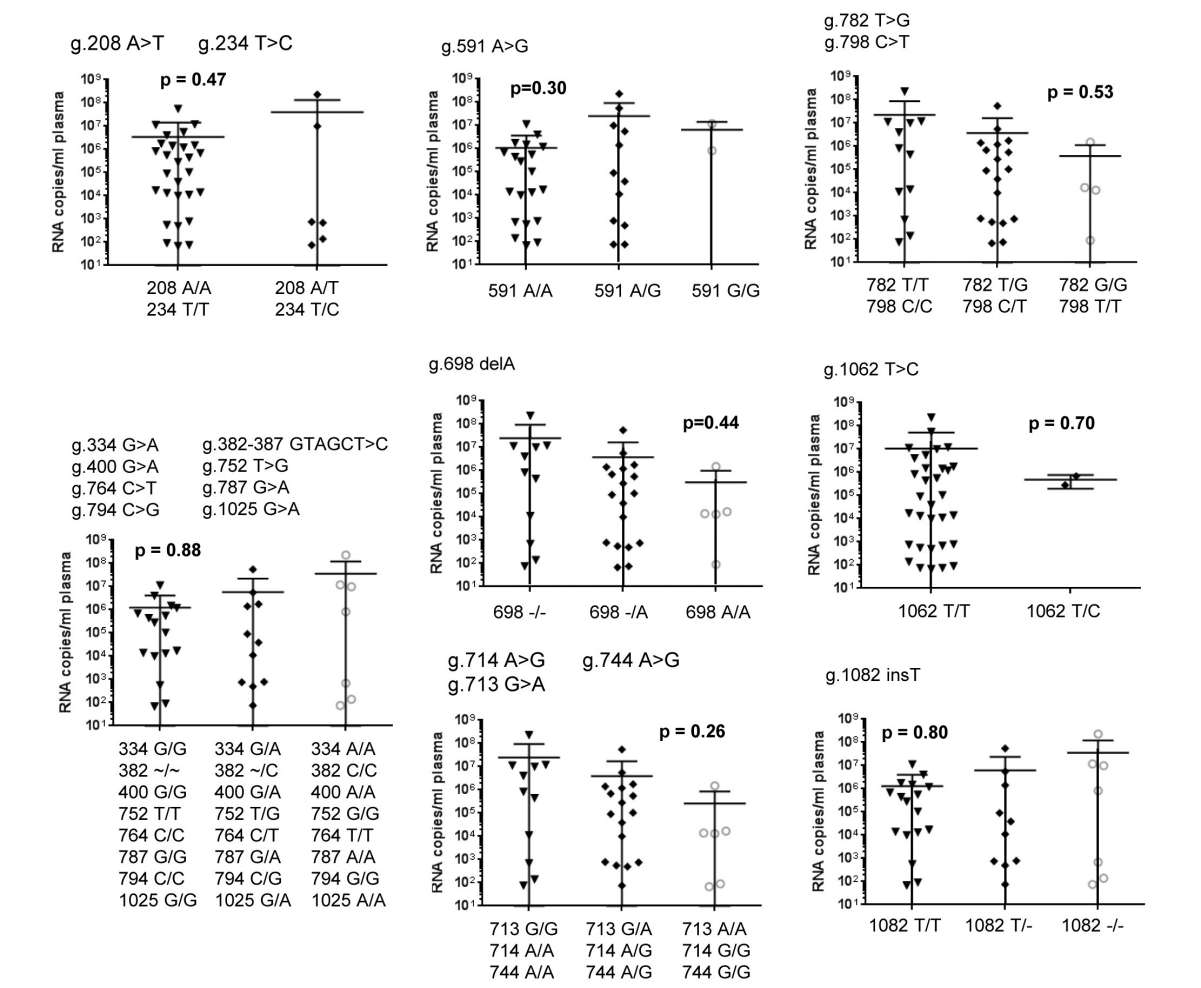
*rhIFITM3(2)* alleles and viral load at set point in SIV-infected rhesus macaques. For each polymorphism, the virus load at set point (20 weeks post infection) was compared between different genotypes. The polymorphisms are indicated above each diagram together with the p-values obtained by a Mann-Whitney or a Kruskal-Wallis test. Individual genotypes are noted below each diagram. Virus load is shown as RNA copies/mL plasma.

Finally, we tested whether the polymorphisms in *rhIFITM3(2)* had an impact on AIDS-free survival time or viral load in SIV infected macaques. However, no statistically significant associations between polymorphisms and AIDS-free survival (Fig 4) or virus load (Fig 5) were detected. We also assessed whether polymorphism 208 A<T, resulting T70S, altered antiviral activity of rhIFITM3(2) in cell culture. However, both rhIFITM3(2)-wt and rhIFITM3(2)-T70S were comparably expressed (Fig 2B) and inhibited MLV-Env, FLUAV-HA and SIV-Env-driven host cell entry with similar efficiency (Fig 2C), although blockade of SIVmac239-316Env-driven entry by rhIFITM3(2)-T70S was somewhat more efficient than that measured for rhIFITM3(2). Thus, T70S may not have an appreciable impact on antiviral activity. Collectively, our results suggest that polymorphisms in the *rhIFITM3(2)* gene do not strongly modulate antiviral activity in cell culture or alter the course of SIV infection in experimentally inoculated macaques.

## Discussion

Human patients harboring a particular polymorphism in the *huIFITM3* gene, rs12252-C, which results in the alteration of a splice acceptor site, were previously shown to be more susceptible to severe influenza than non-carrier patients [10, 36]. They were also found to progress faster towards AIDS [37]. These findings, although not undisputed (see [38–43] for examples), indicates that polymorphisms in *huIFITM3* can impact the course of viral infections. This notion has recently been reinforced by the finding that a polymorphism in the *huIFITM3* promoter that reduces IFITM3 expression increases the risk for severe influenza [44, 45]. Here, we investigated whether rhIFITM3(2) has antiviral activity and whether polymorphisms in *rhIFITM3(2)* impact the course of SIV infection. We showed that rhIFITM3(2) inhibits SIV Env-driven entry in cell culture, although not to the same extent as huIFITM3 and rhIFITM3, and that the *rhIFITM3* gene is polymorphic. Finally, we provided evidence that polymorphisms in *rhIFITM3(2)* do not strongly impact the course of SIV infection.

Expression of huIFITM3 and rhIFITM3 had little impact on entry driven by MLV-Env but markedly inhibited entry driven by FLUAV-HA, as expected [18]. Similar results were obtained for rhIFITM3(2) but inhibition efficiency was lower than with rhIFITM3. Moreover, blockade of SIV Env-driven entry by rhIFITM3(2) was less efficient than that by rhIFITM3. These differences in antiviral activity could be due to reduced expression of rhIFITM3(2) compared to rhIFITM3 However, it should be noted that an IFITM2 specific antibody was used to detect the proteins analyzed here and it is conceivable that amino acid exchanges present in rhIFITM3(2) altered the antibody epitope. In fact, previous analyses conducted with IFITM3 proteins harboring antigenic tags revealed that rhIFITM3(2) is more efficiently expressed than rhIFITM3[27]. Nevertheless, rhIFITM3(2) was less efficient at inhibiting Ebola virus glycoprotein-driven entry than rhIFITM3 [27]. Collectively, these results indicate that rhIFITM3(2) can block viral entry and may thus contribute to inhibition of viral spread in the infected host but its antiviral activity might be lower than that displayed by rhIFITM3.

Our sequence comparisons are based upon rhesus macaque genome scaffold Mmul_051212 [34], which agrees nicely with our previous amplification and cloning of cDNAs [27] and with the analysis of polymorphisms in *rhIFITM3* [18] and *rhIFITM3(2)*, described here. In the more recently described scaffold Mmul_8.0.1 [46] sequences are switched between *rhIFITM3* and *rhIFITM3(2)* in exon 2 and downstream, which we did not observe in our analyses. However, we noted several polymorphisms within the intron sequences that appear to be present in both rhIFITM3 and rhIFITM3(2). As a result, intron sequences from calculated haplotypes of both genes form mixed trees, whereas exon sequences cluster with their respective reference gene. We regard it as unlikely that this mixing is due to PCR artifacts, because additional gene-specific polymorphisms are interspersed between, or close to, these shared polymorphisms.

In a recent study, Sharma and colleagues characterized macaque IFITM genes using evolutionary- and genomic-based approaches [47]. They reported the following gene order for the IFITM locus on chromosome 14: *IFITM5*, *IFITM3A*, *IFITM1*, *IFITM3* and *IFITM3*- pseudogene. This order agrees with work by Compton and colleagues [13] and with our own findings [18]. In addition, we noted that *IFITM3A* and *rhIFITM3*(2) exhibit a similar genomic localization. However, at the DNA sequence level, the 3’ part (including exon 2) of *rhIFITM3(2)* (based upon MmuI_051212) and *IFITM3A* (based upon MmuI_8.0.1, used by Sharma et al) differ by 12 nucleotides within exon 2 (all distinct from the polymorphisms described here). Sequence alignments indicate that in MmuI_8.0.1 the 3’ part of *rhIFITM3(2)* (based upon MmuI_051212) has been exchanged with *rhIFITM3* (based on MmuI_051212). However, our 3’-primer MamuIFITM3(2)gen3 would not be able to bind to this sequence (<65% sequence identity). Therefore, we think that the *IFITM*-encoding region on chromosome 14 has not been fully resolved yet and that our experimental results agree best with the original Mmul_051212 assembly.

The sequence identity of 96% (within the amplified region) between *rhIFITM3* and *rhIFITM3(2)* and the presence of an *IFITM3* pseudogene in the *IFITM* locus on chromosome 14 and additional pseudogenes on other chromosomes highlight that PCR conditions have to be carefully chosen for a specific amplification of *rhIFITM3(2)*. In our previous report, we documented conditions for the specific amplification of *rhIFITM3* that relied upon high annealing temperature (68°C) and the exclusion of pseudogenes with a different size than *rhIFITM3*. These conditions served as a basis for the establishment of the *rhIFITM3(2)* specific PCR used here, of which the 3’ primer, and for some reactions also the 5’ primer, were adjusted to the *rhIFITM3(2)* sequence. A side by side comparison of our *rhIFITM3* and *rhIFITM3(2)* PCRs confirmed our previously reported *rhIFITM3* sequences. Moreover, the sequences amplified by our *rhIFITM3(2)*-specific PCR were different from those obtained with the *rhIFITM3* specific PCR and matched those documented for *rhIFITM3(2)*, confirming that our PCRs were gene-specific.

As for rhIFITM3, the *rhIFITM3(2)* gene was found to be polymorphic and a total of 19 polymorphisms were observed in both exons and introns. Some 16 of the 19 sites were highly polymorphic with a major allele frequency (MAF) of ≤ 75% and homozygous carriers of both alleles were detected. Thus, *rhIFITM3(2)* is more polymorphic than *rhIFITM3*, for which 16 polymorphisms were identified of which only six had a MAF ≤ 75%. On the other hand, many polymorphisms of *rhIFITM3(2)* co-segregate, thereby yielding identical results in the survival and virus load analyses. Of the 19 polymorphisms identified in *rhIFITM3(2)*, 14 were intronic and may thus not affect gene expression. However, an impact of these polymorphisms on RNA stability and pre-mRNA splicing cannot be excluded [48]. In this context, it is noteworthy that none of the polymorphisms altered a splice donor or acceptor sites or a branch point. Five polymorphisms were exonic and two were located in the coding region. One was silent but could nevertheless impact the efficiency of translation and protein folding [49]. The other polymorphism, c.208 A>T, resulted in an amino acid substitution, T70S, and had a MAF of 90%. The T70S exchange is located close to the intramembrane domain 1, the integrity of which has been found to be important for the anti-FLUAV activity of huIFITM3 [8]. However, substitution T70S did not impact expression and antiviral activity of rhIFITM3(2), at least not in transfected cells.

The impact of the rhIFITM3(2) polymorphisms on the course of SIV infection was analyzed using a well characterized animal cohort that previously allowed detection of an association between *TLR7* polymorphisms and survival time in the context of SIV infection [31]. Analysis of these samples revealed that none of the polymorphisms in *rhIFITM3(2)* identified in the present study was significantly associated with time to AIDS development or viral load. We cannot exclude that the analysis of a much larger sample size would reveal such associations. However, the impact of the respective polymorphisms on SIV infection is likely to be low and does not appear to appreciably alter the outcome of experimental SIV infection of macaques. In this context it should be noted that all our analyses were conducted with SIV from macaques (SIVmac), which is highly adapted to macaques. It is still possible that non-macaque-adapted SIVsm (SIV from sooty mangabeys, the precursor of SIVmac) is highly sensitive to rhIFITM3 and/or rhIFITM3(2) and that these factors limit cross-species transmission–a scenario that has been described before for TRIM5α [50] and that warrants investigation in the case of the IFITM proteins as well. Collectively, this and previous studies suggest that changes in the sequences of rhIFITM3 and rhIFITM3(2) are rare and, if they occur at all, do not interfere strongly with antiviral activity, in keeping with the concept that *IFITM1* and *IFITM3* genes in non-human primates underwent purifying selection [51].

## Supporting information

S1 Fig Original uncropped gel images of Fig 2B. (PDF)
