# Supplementary material for: Role of rhesus macaque IFITM3(2) in simian immunodeficiency virus infection of macaques
Source: PLoS One. 2019 Nov 4;14(11):e0224082. doi: 10.1371/journal.pone.0224082 (PMC6827983; doi:10.1371/journal.pone.0224082)
Supplement: S1 Fig — (PDF) [file pone.0224082.s001.pdf]

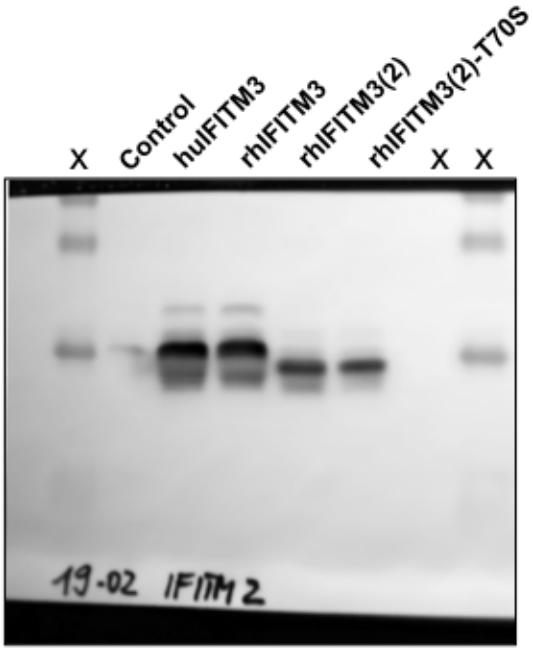

Figure 2B, upper panel
Original, non-cropped image

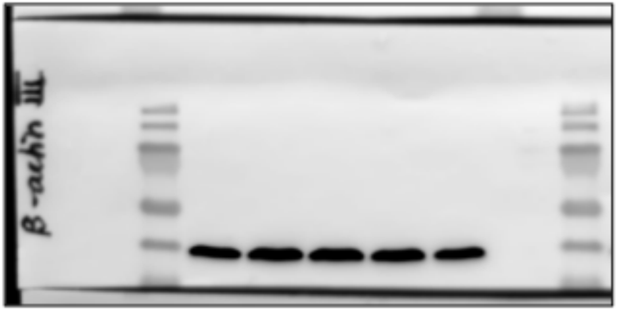

Figure 2B, lower panel
Original, non-cropped image
